# Thymoquinone ameliorates *Pachycondyla sennaarensis* venom-induced acute toxic shock in male rats

**DOI:** 10.1186/s40360-019-0375-x

**Published:** 2019-12-17

**Authors:** Ibrahim M. Alhazza, Hossam Ebaid, Bahaa Abdel-Salam, Jameel H. Al-Tamimi, Iftekhar Hassan, Ahmed M. Rady, Ashraf M. A. Mashaly

**Affiliations:** 1 Department of Zoology, College of Science, King Saud University, P.O. Box 2455, Riyadh, 11451 Saudi Arabia; 2 Department of Biology, College of Science and Humanities in El-Quwiaya, 11961, Shaqra University, Shaqra, Saudi Arabia

**Keywords:** Samsum ant venom, Allergy, PMNs, MHC class II, CD80, CD86, INF-γ, IL-17

## Abstract

**Background:**

For many decades, the sting of Samsun ant (*Pachycondyla sennaarensis*) has been a serious clinical challenge for the people living in some of the major Middle East and Asian countries. In the present study, the therapeutic potential of *Nigella sativa* derived plant extract component, thymoquinone (TQ) has been tested against the Samsun ant venom (SAV) at the toxic dose in the rats.

**Methods:**

The adult male rats were divided into four groups (*n* = 10): control, SAV treated, SAV + TQ treated and TQ alone treated. It was found that the sub-lethal dose of SAV alters not only many of the kidney and liver function markers but also induces oxidative stress in the animals. Moreover, the SAV also disturbs various immunological parameters including expression of PMNs, CD-80, CD-86, interleukins and other cytokines compromising the affected organism towards mild to severe allergic reactions including life-risking anaphylaxis.

**Results:**

The plant extract, TQ, effectively restores many of the biochemical and oxidative stress parameters comparable to the normal concomitant with improving the immunological aspects that might attributive in relieving from SAV-induced toxicity and allergic reactions in the affected organism to a greater extent.

**Conclusion:**

Hence, TQ has an excellent antidote property against SAV-induced toxicities in vivo. Although the study is a vivid indication of the potential therapeutic potential of TQ against the SAV induced in vivo toxicity, yet the actual mechanism of interaction translating the toxicity amelioration warrants further investigations.

## Background

The Samsun ant (*Pachycondyla sennaarensis*) is one of the most extensively studied insects in recent times. The Samsun ant venom (SAV) has been found responsible for intense toxic including the cases of anaphylaxis across the globe. The venom produced by such ants are abundant in diverse enzymatic and non-enzymatic proteins, free amino acids, and small biologically active compounds [1]. Recent studies reveal that either the whole venom at specific doses or some of its bioactive chemical compounds are promising therapeutic candidates against various diseases and allergies [2, 3]. Furthermore, Sousa et al. [4] have demonstrated the inflammatory effect of *Dinoponera quadriceps* venom characterized by edema and enhanced vascular permeability concomitant with neutrophil migration implying the participation of resident macrophages and IL-1β in a rodent-based study. Also, Lima et al. [5] recently reported a strong antimicrobial effect of SAV on the parasites. Hence, the venom has a reservoir of significant untapped leads of novel therapeutic and bio-insecticide potentials [2].

In general, human toxicity caused by these ant venoms results in pain, local inflammation, itching, and irritation, while sometimes they lead to serious allergic reactions including severe anaphylactic shock triggered by a structurally diverse mixture of their component compounds [6, 7]. Hence, the sting of such threatening ants is posing a significant public health hazard in their target habitats including Saudi Arabia, the Middle East, and some Asian countries. Similarly, the jumper ant (*Myrmecia pilosula*) in Australia whose venom rich is in the polypeptides Myr p 1, and Myr p 2 has been reported for causing severe allergic reactions in the sensitive individuals [8–10]. Moreover, the sting of *Hymenoptera* also leads to a mild local reaction with painful erythematous swelling or even severe life-threating anaphylaxis [11]. In all these cases of venom shock, venom immunotherapy has been found a highly effective treatment [12].

On the other hand, a large number of medicinal plants and their purified constituents as potential therapeutic agents nowadays drawing research-interests among many contemporary researchers. Herbs-based medicines being safer than the ones of the commercial drug depends on their mode of administration, the patient’s history, and their specific disease conditions [13, 14]. As plant extract-based herbal medicines are a mixture of complex constituent compounds and bioactive elements, their mechanism of action is quite different although less invasive as compared to the allopathic counterfeit drugs comprised of well-defined ingredient molecules [15, 16]. Lots of literature entails that *Nigella sativa* has tremendous healing properties against various types of wounds with curing and relieving properties in a wide range of diseases and allergies [17]. *It is widely documented that N. sativa* has a broad array of biological activities including a diuretic, antihypertensive, antioxidant, analgesic, antimicrobial, anthelmintic, analgesics and anti-inflammatory, spasmolytic, bronchodilator and immunomodulatory properties. All these pharmacological properties make this plant clinically significant for possible treatment of serious diseases like diabetes, pulmonary issues, general chemicals mediated toxicity and even cancer [18]. Also, it is also documented that the presence of thymoquinone (TQ) as a primary bioactive component in its extract is responsible for most of the biological and pharmacological effects [19].

The present investigation is aimed at discoursing the immunological, histological and biochemical alterations post SAV treatment at relatively high doses (600 μg/Kg) in rats as an animal model system. Furthermore, we were also interested to investigate if TQ is effective in alleviating these SAV-induced in vivo aberrations.

## Methods

### Chemicals and reagents

Succinic acid, potassium dihydrogen and mono-hydrogen phosphate, glycine, pyrogallol, hydrogen peroxide, trichloroacetic acid (TCA) and ethylenediaminetetraacetic acid (EDTA) sulphosalicylic acid and thiobarbituric acid (TBA) were purchased from BDH, England. 5,5-dithiobis-2-nitrobenzoic acid (DTNB), dihydrogen phosphate, trichloroacetic acid, carbon tetrachloride, and thiobarbituric acid were obtained from Merck Company, Darmstadt (Germany). Rest all other chemicals including carbon tetrachloride (CCl_4_) were from Sigma-Aldrich Company (St. Louis. MO, USA). Thymoquinone (TQ) was naturally derived from the *Nigella sativa* leaves extract in the present study.

### Collection of the samsum ant venom

Samsum ant colonies were collected from Hotat Bani Tamim Governorate, East Riyadh, Kingdom of Saudi Arabia. The extracted dirt block containing the nest of ants in suitable cloth bags were moved to the ant insectary in the Department of Zoology, King Saud University. Further, they were housed in specially designed plastic containers (volume capacity of 20 × 70 cm) with painted upper interior edges by grease to avoid any possible exit of ants. They were supplied a glass tube containing sugar solution (10% w/v). Moreover, they were fed with wheat grains crushed or pills tile of the nest spray volumes of water twice a day according to the standard ant harvesting method with maintained hygiene. The venom gland from the insects was separated by grabbing in the area of pregnancy with the forceps gently. Then, the venom glands were homogenized followed by their centrifugation at 1000 rpm for 2 min. And thus the supernatant was collected in the Eppendorf microfuge tubes stored at − 25 °C in PBS buffer until use.

### Experimental design

Sixty-four Wister male rats weighing 220–270 g (aged at 20 ± 1 weeks) were purchased from the Central Animal House facility in the Faculty of Pharmacy, King Saud University, Riyadh (Saudi Arabia). The animals were acclimated to the departmental animal house (Department of Zoology, KSU) conditions for two weeks before the starting of the treatment. They were housed in stainless steel wire cages (6 animals/ cage) under pathogen-free conditions at pellet diet, and fresh tap water *ad labium* with the temperature maintained at 22 ± 2 °C with a relative humidity of 45–65% and day/night cycle of 12/12 h. All the animals were weighed before dosing and were under keen observation for signs of ill health. The animal handling and treatment methods were duly approved by the Animal Ethical Committee of the Zoology Department, College of Science, King Saud University (Riyadh).

The rats were randomly divided into four groups (*n* = 8). The first group was the control without any treatment while the second group was treated with the SAV (600 micrograms/kg) via intraperitoneal. The third group was treated with SAV (600 micrograms/kg) followed by TQ (20 mg/kg) at the gap of 30 min. The fourth group was treated with TQ (20 mg/kg) alone. The dosing in second, third and fourth groups was administered twice a week and the whole treatment duration was three weeks. At the end of the treatment, the average body weight (in gram) of CN, SAV and SAV + TQ was found 322.08 ± 27.70, 168.23 ± 15.42 and 261.91 ± 9.51 g respectively.

### Sacrifice of the animals and sample collection

After completion of the duration of the treatment, all the animals were sacrificed by ether anesthesia. The anesthesia of rats was strictly conducted by the expert personnel with all International safety measures framed for ether usage and disposal as per guidelines of Animal care and use committee of Johns Hopkins University (USA) with all required permissions. After reaching successfully maintained anesthesia by ether, the blood (maximally ~ 7 ml) was withdrawn from the animals. After the withdrawal of blood, the rats were very calm as they were undergoing slow death, which allowed us to excise the target organ, liver from them. Blood was collected in two separate tubes having EDTA as an anticoagulant for each animal. Besides, the liver from the animals was rinsed with BPS (7.4) to eliminate blood contamination followed by drying with blotting filter paper. All the samples were finally stored in BPS in a deep freezer (− 80 °C) until further analysis directly.

### Sample preparation and assessment of HDL and cholesterol

The sera were separated by centrifugation of blood from each animal at 3000 rpm for 10 min that were stored at − 25 °C. All the liver samples perfused with PBS (pH 7.36) at 10% (w/v) were homogenized using a homogenizer; from which the respective supernatants were obtained by centrifugation at 10000×g for 15 min at 4 °C, and kept in separate glass vials at − 25 °C.

The level of HDL and cholesterol was chosen under this part of the study using commercially available kits (QCA, Spain). The triglycerides concentration was determined calorimetrically using the commercial kit (Salucea Company, Netherlands).

### Assay of serum markers

The sera samples were subjected to estimation of AST, ALT, ALP, creatinine and urea to assess liver and renal function using commercial kits (Quimica Clinica Aplicada S.A., Spain) by Spectrophotometric method (Pharmacia Biotech, Ultrospec 2000 model, Cambridge, England).

### Assessment of GSH and MDA level

The level of reduced glutathione (GSH) was estimated by the method of Jollow et al. [20] while the malondialdehyde (MDA) was determined according to the method by Ohkawa et al. [21].

### ELISA for IFN-γ and IL-17

The liver homogenate was assayed for IFN-γ and IL-17 by ELISA kits (MyBioSource and Ray biotic, US) according to the manufacturer’s instructions. The plates were measured at 450 nm (Anthos 2020, UK). The detection limits were set as per the log-log correlative coefficient of the standard curve.

### Immunofluorescent staining and FACS analysis

For fluorescence-activated cell sorter (FACS) analysis of whole blood, erythrocyte FACS lysing solution was obtained from Becton Dickinson (Heidelberg, Germany) that was diluted 1:10 with double distilled water. For cytofluorometry fluorescein isothiocyanate (FITC) and phycoerythrin (PE) - labeled murine monoclonal Abs were used. MHC-DP + DQ + DR: PE, CD80:FITC and CD86:PE were obtained from Coulter Immunotech (Marseille, France). Cells in whole blood were analyzed by FACS Calibur and Cell Quest software (Becton Dickinson, SanDiego, CA, USA). In the present study, expression of MHC class II and CD80 and 86 was conducted by the mentioned method. Results were expressed as the percentage of positive cells in the respective gate. In FACS plots, there were different populations; due to which the gates were made around the population of PMNs.

### Statistical analysis

The statistical analysis of the data was conducted by one-way ANOVA, and the data were normally distributed with homogeneous variances. The results were expressed as the mean (M) ± standard deviation (SD). Only statistically significant differences were set at *P* < 0.05 between the treatment groups and the control. Minor fluctuations were observed upon the repetition of most of the experiments in a blindfolded manner. These deviations are evident from the figures of the present study.

## Results

In this study, we investigated the biochemical and immunological changes after the treatment on the animals. The biochemical analysis was aimed to observe the effect of present therapy on the lipidogram, the level of lipid peroxidation and the reduced glutathione besides primary serum markers of liver and kidney function test in the animal samples. Moreover, the samples were investigated for the immunological changes based on modulation of MHC-Class II, CD-80, and CD-86 with an expression of PMNs.

### The effect on the lipid peroxidation

The administration of SAV led to increases in TAGs by 10.93% as compared to the control while SAV+ TQ treated group showed an increase of 24.03% as compared to the SAV treated group. The venom was found to substantially increase the lipid peroxidation in both the organs: liver and kidney by staggering 848 and 74.5% respectively. Interestingly, TQ in the SAV-pretreated group sharply decreased lipid peroxidation by 92.82, and 23.59% in the liver and kidney samples (Table 1). It entails that TQ is more effective in healing SAV-induced hepatotoxicity as compared to renal toxicity.

**Table 1:** Effect of TQ on the concentrations of oxidative stress parameters induced in the different groups

Biochemical Parameters	Control	SAV	SAV + TQ
GSH in liver (nmol/mg protein)	6.34 ± 0.4	3.74 ± 0.2*	4.25 ± 0.21
GSH in kidney (nmol/mg protein)	6.05 ± 0.6	4.11 ± 0.11*	4.89 ± 0.23*
MDA in liver (nmol/mg protein)	0.25 ± 0.1	2.37 ± 0.4	0.17 ± 0.08
MDA in kidney (nmol/mg protein)	0.51 ± 0.2	0.89 ± 0.5*	0.68 ± 0.5

### The effect on the glutathione

The treatment of TQ in the SAV-pretreated group led to improving the GSH level moderately as compared to SAV alone group in liver and kidney samples by 13.63 and 18.97% respectively (Table 1).

### The effect of the treatment on the lipidogram

In the present study, SAV caused a significant decline by 54.75% in the concentration of high-density lipoprotein (HDL) as compared to the control. However, the treatment with TQ led to replenish the HDL level better than the control. The cholesterol level was also enhanced in the SAV-treated group by 100% which was found to decline in the SAV and TQ group to a significant extent by 46.36%. (Table 2).

**Table 2:** Effect of TQ on the concentrations of lipidogram, liver and kidney functions, and IFN-γ induced in the different groups

Parameters	Control	SAV	SAV + TQ
Triglycerides (TAGs) (mg/100 ml)	72.67 ± 5.61	80.62 ± 6*	100 ± 11.2*
Cholesterol (mg/100 ml)	85.27 ± 5.8	170.54 ± 22.9*	91.47 ± 18.3
High density lipids (HDL) (mg/100 ml)	13.46 ± 2.2	6.093*	25.50 ± 3.7
ALT (U/L)	69.11 ± 9.3	69.91 ± 6.6	68.52 ± 6.91
AST (U/L)	70.06 ± 4.7	69.96 ± 6.5	68.52 ± 7.11
ALP (U/L)	129.90 ± 6	135.61 ± 5*	124.43 ± 10
Urea (mg/100 ml)	56.94 ± 7.2	96.94 ± 5.7*	40.78 ± 3.2
Creatinine (mg/dl)	0.52 ± 0.0412	0.56 ± 0.0202	0.61 ± 0.061
IFN-γ (pg/ ml)	269 ± 11	153.5 ± 6*	261 ± 13
IL-17 (pg/ ml)	684 ± 34	450 ± 26*	605 ± 33.9

### Effect on the liver and kidney functions

The major liver function marker enzymes, ALP was elevated in the SAV group by 4.39%, while, the plant extract caused in its decline towards the control level by 8.24% (Table 2). The SAV-treated group demonstrated an elevated level of urea by 70.25% while TQ caused a significant decrease in its level the in SAV pretreated group by 57.93%. No significant changes were recorded in the levels of AST, ALT and creatinine (Table 2).

### Effect on the immunological parameters

The second experimental sets testing the modulation effect of plant extract on the expression of MHC-Class II, CD-80 and CD-86, are as follows:

#### Modulation of MHC-class II expression on PMNs by plant extract

**Fig. 1:**
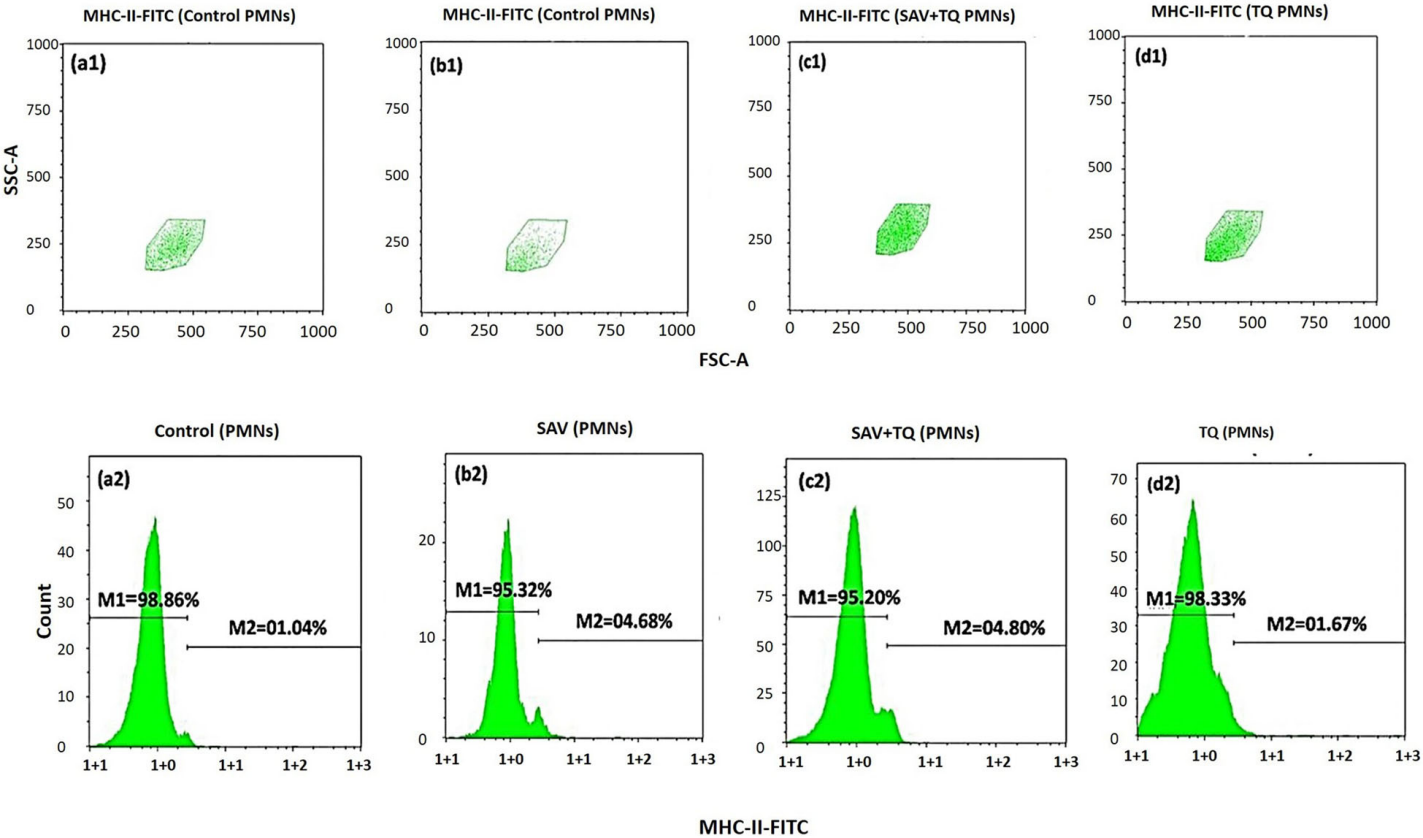
Cytofluorometry of the MHC class II induction and modulation in the whole blood PMNs. (a1&2) Unstimulated PMNs (control). (b1&2) SAV stimulated PMNs. (c1&2) SAV + P2 stimulated PMNs. (d1&2) P2 treated PMNs

In this set of experiments, we tested the expression of MHC-II on the respective gate of PMNs. The control group showed a change in its expression by 1.04% (Fig. 1a2) while SAV-treated group showed an increase in its level by 4.68% (Fig. 1b2). However, SAV + PE demonstrated 4.80% of the growth in the expression (Fig. 1c2) while the PE alone showed a similar reading of 1.67% change (Fig. 1d2). Figure 1a1, b1, c1 & d1 indicated the FSCA/SSCA plot of gated PMNs.

#### Modulation of CD-80 expression on PMNs by plant extract

**Fig. 2:**
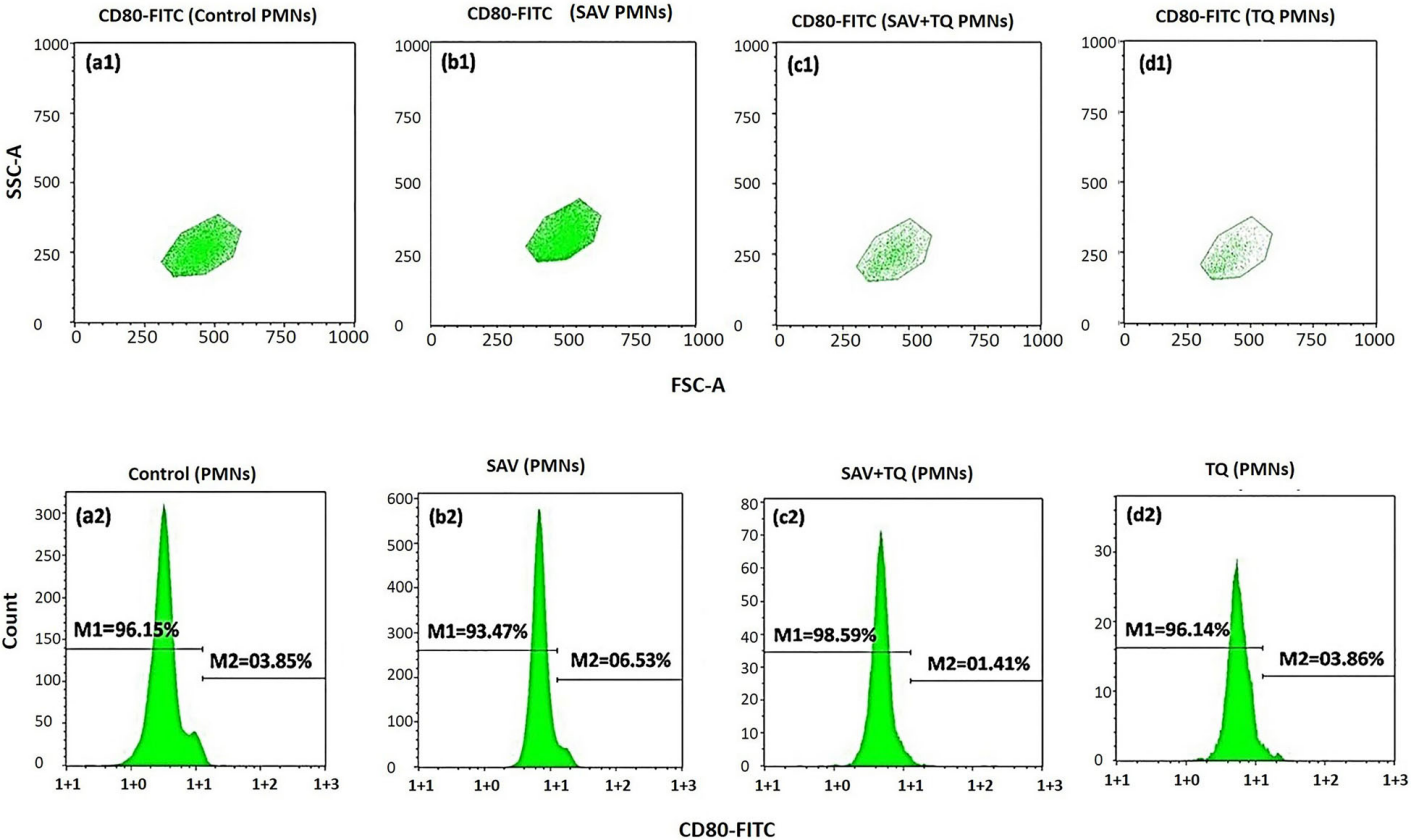
Cytofluorometry of the modulation of the CD80 induction on PMNs in whole blood. (a1&2) Unstimulated PMNs (control). (b1&2) SAV stimulated PMNs. (c1&2) SAV + P2 stimulated PMNs. (d1&2) P2 treated PMNs

In this set of experiments, we tested the expression of CD80 on the respective gate of PMNs. The SAV injected PMNs in Fig. 2b2 showed an increase in CD80 expression showing recording as 6.53% as compared to the control and the plant extract; P2 treated PMNs was found counting at 3.85% (Fig. 2a2) and 03.86% (Fig. 2d2). The SAV + P2 treated PMNs showed a little amount of change by 1.41% (Fig. 2c2). Figure 2a1, b1, c1 d1 showed the FSCA/SSCA plot of gated PMNs.

#### Modulation of CD-86 expression on PMNs by plant extract

**Fig. 3:**
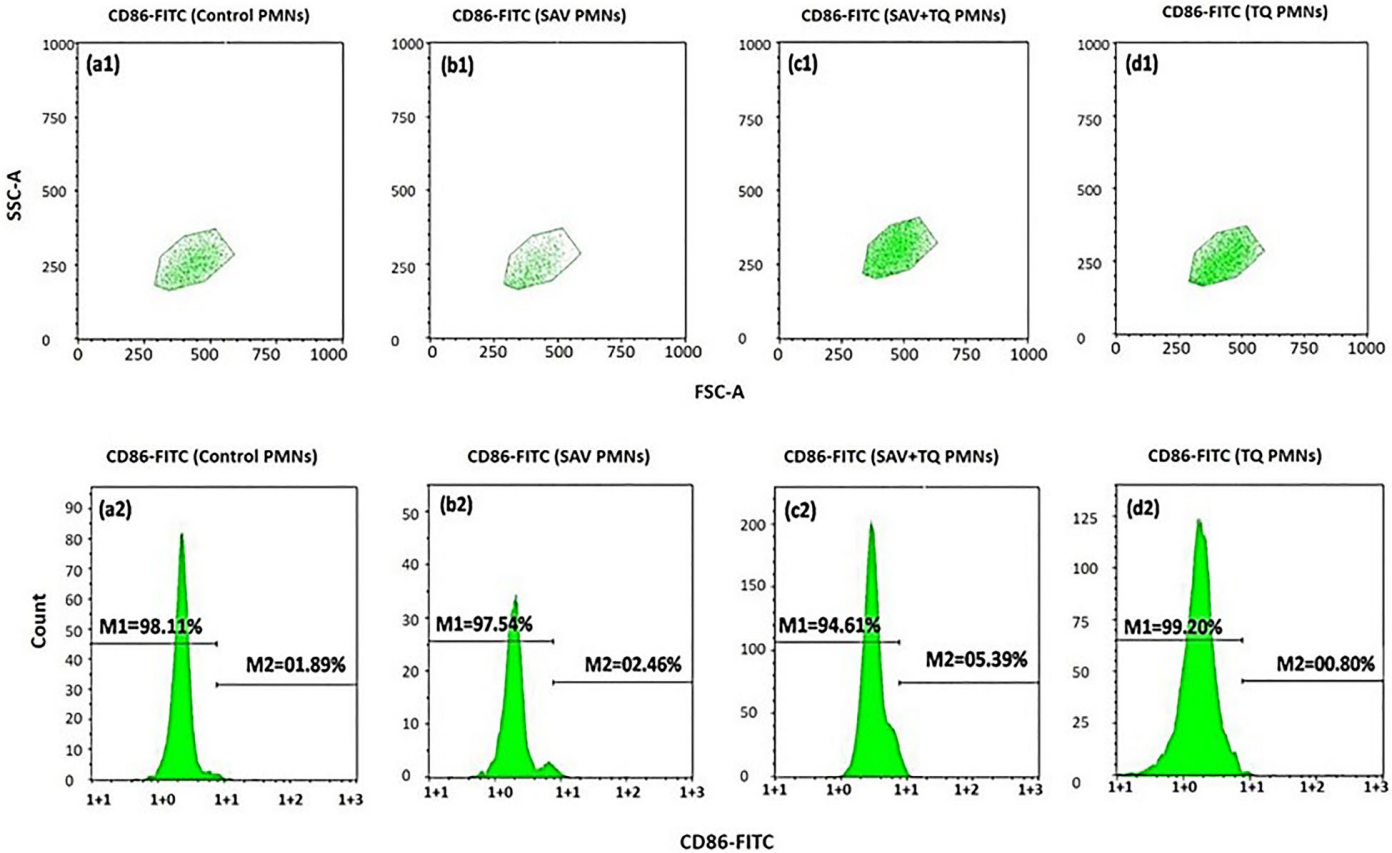
Cytofluorometry of the effect of P2 on CD86 induction in the whole blood PMNs. (a1&2) Unstimulated PMNs (control). (b1&2) SAV stimulated PMNs. (c1&2) SAV + P2 stimulated PMNs. (d1&2) P2 treated PMNs

Figure 3a2 illustrates the expression of CD86 on the PMNs respective gate the control group (1.89%) which was lower than both the SAV injected group (2.46%) and the SAV + P2 treated group (5.39%), Fig. 3b2 and c2 respectively. P2 treated group (00.80) is nearly similar to the control (Fig. 3d2). The FSCA/SSCA plot of gated PMNs has been shown in Fig. 3a1, b1, c1 & d1).

#### Effect of IFN-γ

The treatment with SAV led to decrease in this parameter by 42.93% as compared with the control. However, SAV + TQ treated group showed replenishment in its level by 70.03% concerning SAV group (Table 2).

#### Effect of IL-17

The SAV treatment led to a decrease in this parameter by 34.21% as compared with the control. However, SAV + TQ treated group showed replenishment in its level by 34.44% with respect to the SAV group (Table 2).

## Discussion

Ants represent a taxonomically diverse group of hymenopterans with over 13,000 extant species. The majority of them secrete venom from the venom gland that assists them in preying and also provides safety [22]. Animal venoms are complex mixtures of a wide range of bioactive elements including polycyclic hydrocarbons, catecholamine, histamine, and dopamine [3]. We have found that SAV, at a low dose, is a promising source that offers potential biologically active properties [1, 23, 24] that may be useful as new tools for the design of drugs. Sousa et al. [4] demonstrated that the inflammatory effect of venom of *Dinoponera quadriceps* characterized by edema, an increase in vascular permeability and neutrophil migration that implies the participation of resident macrophages and IL-1β, among other inflammatory mediators. Similarly, SAV is an important health concern in the habitats including Saudi Arabia, Middle East, and some Asian countries. The sting of Samsum ant causes mild to severe toxic effects in the affected individual including pain, local inflammation, edema, and erythema. Moreover, the sting can also lead to severe anaphylaxis depending on individual’s hypersensitivity. Despite the widely studied effects of this venom, the exact mechanism of action in vivo is yet to be elucidated that warrants further research [11].

On the other hand, a large number of medicinal plants and their purified constituents have shown beneficial therapeutic possibilities. In the present paper, we applaud the plant extract of *N. sativa* as herb treatment against the SAV- induced effects in the animal model study. The extract of *N. sativa* has established bioactive and immunostimulant components with potentials including blunting of serious post-sting complications to nullify the effects of adverse reactions of various toxins [25]. Therefore, the present work explores the biochemical, and immunological alterations post high dose of SAV in the rodents, followed by confirming the ameliorative potential of the plant extract if it can restore all the studied parameters towards normalcy in vivo.

SAV-treated rats in the present study exhibited a profound elevation in total cholesterol and LDL-cholesterol levels concerning the control. These results entail that high dose of SAV can trigger cardiovascular risk indices represented by the ratios of the total cholesterol levels and LDL-cholesterol to HDL-cholesterol levels. Interestingly, the treatment of the plant extract in pre-SAV-dosed rats showed a marked decline in these perturbed serum lipids. Also, MDA levels and GSH, the two most reliable indicators of oxidative stress [14] were significantly altered in SAV- treated rats compared with the corresponding control animals in the liver samples. These results are in agreement with the previous studies [1, 26] suggesting that oxidative stress regardless of its source, induces cellular dysfunction in endothelial and smooth muscle cells leading to a reduction in angiogenesis and the healing process. However, on the other hand, the administration of SAV- treated rats with the plant extract profoundly decreased the MDA levels concomitant with improved the antioxidant defense system by demonstrating significantly increased hepatic glutathione levels. These findings also agree with improved renal and liver function markers to a significant extent. These findings indicate that the plant extract may restore the tissue, at least in part, by the modulation of the antioxidant defense system. However, there was a prominent discrepancy in the SAV-induced toxicity and ameliorative effect of TQ in liver and kidney samples. It infers that the mechanism of action of both the treatment solutions is different in liver and kidney that might be attributive in the differential efficacy of both the test compounds in the rodents. Further studies are underway to investigate this aspect of the study.

The immunological part of the present study entails that stimulated PMNs by SAV led to the expression of the antigen-presenting molecules (MHC-II) and the co-stimulatory molecules (CD80 and CD86) under the effect of the regulation of some cytokines such as INF-γ and IL-17. We detected high levels of both INF-γ and IL-17 in the sera of SAV-treated animals. Hence, SAV-stimulated PMN might be involved indirectly in acquired immune response in addition to their role in innate immunity. So, the data confirm this, where the proponents found the expression of MHC-II, CD80, and CD86 on stimulated PMNs.

We hypothesized that SAV might induce acute toxic inflammation on the taken dose leading to initiation and activation of PMNs. Also, we tested an active gradient from a medicinal plant, TQ to testify if it restores PMNs surface-co-stimulatory molecules. Under certain stimulation murine neutrophils present MHC-II restricted antigen [27–29]. The current work was aimed to estimate the expression of MHC-II along with CD80, and CD86 on PMNs stimulated with SAV. It is well known that MHC-II and CD80 and CD86 play an important role in T-cells proliferation, where MHC-II presents the engulfed antigen on the T-cells [29] and CD80 and CD86 involved in the stimulation of T-cells to produce IL-2 that are essential for T-cells proliferation [30]. Additionally, the activation and recruitment of PMN are also regulated by IL-15, IFN-γ and IL-8 [31–33].

Recent literature suggests that the mechanism of the anti-inflammatory action in the butanolic fraction of Byrsonima verbascifolia leaves is linked to the inhibition of the production of inflammatory mediators such as TNF-α and PGE2 and the PMN cell migration [34, 35]. Sadeghi et al. [36] confirmed an anti-edematogenic activity of maprotiline in the carrageenan-induced paw edema model showing such properties of maprotiline might be mediated through inhibition of PMN infiltration and release of IL-1β and TNF-α. Furthermore, the present study agrees with Hassan et al. [37, 38] who have pleaded that regulation of oxidative stress is critical for ceasing of any toxicant mediated inflammation and resulting in oxidative damage in vivo.

## Conclusion

**Fig. 4:**
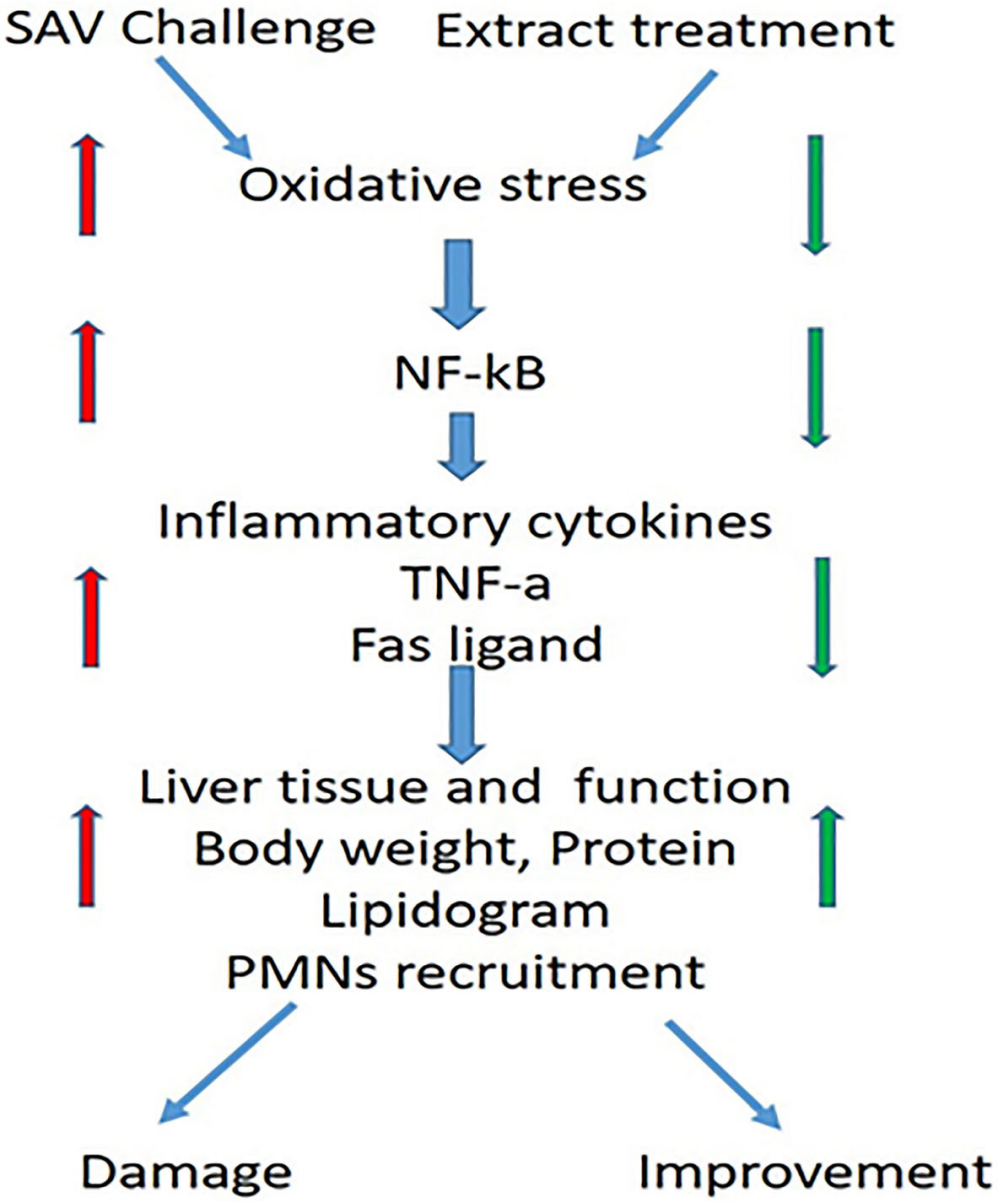
Diagrammatic representation of the putative mechanism involving the major events after challenge with high doses of SAV and the treatment with the selected plant extract

The present study brightly shows that TQ is a pharmacologically useful compound (Fig. 4) which can restore the SAV-induced redox and biochemical alterations to the normal concomitant with modulating many critical immunological parameters in vivo. Additionally, our findings describe a potential role for CD80 and CD86 in the regulation of the innate immune response during SAV-induced inflammation. Many investigators are nowadays working on targeting the receptors capable of simultaneously regulating numerous pro-inflammatory cytokines such as CD80 and CD86 to contain inflammation for better treatment of inflammation-mediated diseases. Hence, the present study entails the promising therapeutic strategy of TQ to counter SAV-induced inflammation by modulating the direct inhibition of CD80 and CD86.

## Data Availability

The relevant data to work have been included in the paper. However, the supplementary data may be shared on request to the main contributing authors on request.

## Abbreviations

IL: Interleukin IFN-γ Interferon-γ HLA-II Human leukocyte antigen class II MHC-II Major histocompatibility class II PMNs Polymorphonuclear neutrophils
